# Poetry as a Creative Practice to Enhance Engagement and Learning in Conservation Science

**DOI:** 10.1093/biosci/biy105

**Published:** 2018-10-04

**Authors:** Stephanie R Januchowski-Hartley, Natalie Sopinka, Bethann G Merkle, Christina Lux, Anna Zivian, Patrick Goff, Samantha Oester

**Affiliations:** 1 Department of Biosciences at Swansea University, in Swansea, United Kingdom; 2 Canadian Science Publishing, in Ottawa, Ontario; 3 Wyoming Migration Initiative, in the Department of Zoology and Physiology at the University of Wyoming, in Laramie; 4 Center for the Humanities at the University of California, Merced; 5 Ocean Conservancy, in Santa Cruz, California; 6 Beaumont Middle School, in Beaumont, Kentucky; 7 Environmental Science and Policy Department at George Mason University, in Fairfax, Virginia

**Keywords:** creativity, communication, education, interdisciplinarity, professional development

## Abstract

Creativity is crucial to the capacity to do science well, to communicate it in compelling ways, and to enhance learning. Creativity can be both practiced and enhanced to strengthen conservation science professionals’ efforts to address global environmental challenges. We explore how poetry is one creative approach that can further conservation scientists’ engagement and learning. We draw on evidence from peer-reviewed literature to illustrate benefits of integrating science and poetry, and to ground our argument for the growth of a science-poetry community to help conservation scientists develop skills in creative practices as a component of professional development. We present examples from literature as well as two short poetry exercises for scientists to draw on when considering writing poetry, or deciding on forms of poetry to include, in their practice. Opportunity exists to grow science–poetry projects to further our understanding of what such initiatives can offer.

Current interdisciplinary dialogue generally perpetuates the ideology that scientists do science and artists do art. However, research and experience shows that scientists—and society more broadly—benefit from scientists creating works beyond their discipline (Swanson et al. 2008, Opermanis et al. 2015). Broadly, *creativity* is defined as the production of original and useful ideas (for a broader discussion of creativity, see Stein 1953, Barron 1955, Runco and Jaeger 2012), and a variety of creative approaches, primarily from the arts, are increasingly appreciated in science education, communication, and practice (Jacobson et al. 2007, Swanson et al. 2008, Opermanis et al. 2015). For example, in Latvia, the Nature Concerthall brought science and arts (poetry, music, dance, photography, and videography) together as part of an information campaign to enhance public knowledge and awareness of nature conservation issues and resulted in both greater attendance and perceived greater knowledge of biodiversity issues by attendees (Opermanis et al. 2015). At the same time, the integration of creative practice in professional development opportunities for scientists is increasing; the last several years have seen multiple speakers at ecology and environmental conservation conferences (e.g., Society for Freshwater Science 2018, World Conference on Marine Biodiversity 2018, Resilience 2017) using different creative practices to highlight the role and value to ecology and environmental conservation of these practices. At the World Conference on Marine Biodiversity 2018, in Montréal, Canada, Linwood Pendleton's plenary, “Rethinking marine conservation science in three acts,” brought together poems, music, video, and dance to demonstrate how creative approaches can help to achieve and celebrate breakthroughs in marine conservation science (Pendleton 2018). Research focused on innovation in science also demonstrates that creativity is something we can practice and improve and that proficiency in a fine art, craft, or literary pursuit is a significant predictor of scientific productivity and innovation (Root-Bernstein 2003). Poetry, the focus of our article, is one creative practice that conservation scientists can use to enhance their capacity to innovate, to communicate their work in compelling ways, and to enhance their own learning, as well as that of others.

We recognize that there is a well-established body of environmental writing; the Association for the Study of Literature and Environment was established in 1992. We also recognize the growing area of environmental humanities research, which is strongly driven by those working in the arts and humanities. People working in environmental humanities are building interdisciplinary collaboration and research and reflecting on and critiquing actions and inactions when it comes to the use and management of our natural world (e.g., Magrane and Johnson 2016). Our article is directed at conservation scientists who have included or who are interested in creating poetry in their practice and are not poetry or literary specialists. The article is directed primarily at conservation science and scientists because of our own backgrounds and experiences, but we draw on examples from and our arguments are applicable to diverse fields. We do not see our article as separate from the ongoing work or research in environmental humanities but complementary to it and supportive of the idea that we require interdisciplinary lenses and creative approaches to action, critique, and reflection when it comes to environmental conservation and sustainability. Indeed, conservation scientists can benefit from engaging the writing and research of the environmental humanities; doing so would reinforce what we present below and would potentially encourage new and broader interdisciplinary research opportunities and directions. In the present article, we encourage a more explicit linking of conservation science and poetry by engaging scientists in poetic practice that can shape their work and believe this goes beyond wilderness literature, nature poems, and ecocriticism to consider how scientists can learn from creative practices in poetry to enhance their scientific practice.

Our focus in this article is on the unity of science and poetry, and we draw from evidence in science education, creativity, and problem-solving literature to demonstrate the potential benefits of science and poetry integration and ways in which conservation scientists can use poetry with their science as a component of professional development. To further illustrate, we reflect on our experiences and provide resources from our own ongoing science-poetry projects. To support a growing science-poetry community, we highlight new approaches of integrating science and poetry that may foster creativity and inspire others to find their own ways of building creative practice in their science.

## Integrating science and poetry: Lessons from the classroom

Across diverse scientific fields, students have expressed a sense of enhanced engagement and enjoyment when poetry is integrated with their core subject. For example, Furlan and colleagues (2007) merged poetry writing and illustration in a college-level chemistry course. Their students noted that including poetry in the assignments not only made chemistry more enjoyable but offered a creative way to learn and communicate with others about chemistry. Similarly, Celly (2009) demonstrated the use of limericks for business management students to develop creative expression and reflect on their experiences as consumers to enhance topic engagement and deepen learning. In both examples, when poetry was integrated with core content, students were more engaged rather than being passive recipients of knowledge or information (Furlan et al. 2007, Paiva et al. 2013). Enhanced engagement can result in a topic or problem being perceived as more enjoyable or accessible, because individuals can more effectively participate, both cognitively and emotionally (Lin et al. 2013).

Integrating poetry with science can also enhance opportunities for communication with others about a subject or problem. To illustrate, in his role as a middle school science teacher, Patrick Goff (article coauthor), searched ways for his students to exercise their creativity during a science module focused on human impacts to Earth's ecosystems. In his search, Goff found several initiatives led by conservation scientists who were using poetry to communicate about the environment and conservation on the social media platform Twitter. Inspired by the various conservation-poetry projects, Goff integrated haiku into his course. He considered poetry writing an opportunity for his students to showcase their creativity while learning about how humans affect Earth's ecosystems, to learn and communicate about these impacts, and to share their emotions about these impacts, beyond the boundaries of traditional pedagogical tools and approaches (e.g., reading from textbooks). In turn, Goff asked his students to select a topic related to human impacts on Earth's ecosystems, research it, and then write a haiku about it. He also shared some of the students’ haiku on Twitter, with their consent, to expand the potential audience reading the poems and to solicit potential feedback for the students. Goff's approach complements a framework suggested by Frye and colleagues (2011) to extend acrostic poetry into different content areas to bring ownership to students’ understanding. Goff found the succinct structure of haiku appealing from an instructional point of view; the short form demonstrated to his students the importance of selective word choice and the value of concentrated reflection on the topic. The students also expressed enjoyment in writing the haiku as an alternative to other modes of writing or assessment and in having their poems shared with others and receiving feedback.

The attitudes expressed by Goff's students about the integration of poetry in their science curriculum align with broader findings demonstrating the impact of topic engagement on learning (Paiva et al. 2013). Social and behavioral science studies have shown, for both children and adults, that activities that generate enjoyment or humor in an educational setting can stimulate learning, because students are more relaxed and less bound by rules (Lucardie 2014). An added benefit of short-form writing such as poetry is the potential to receive relatively rapid feedback when sharing ideas, which is important in learning environments (Hattie and Timperley 2007).

## Integrating science and poetry: Benefits to conservation

Poetry can allow scientists to engage, learn, and generate new ideas by enabling them to gain distance from an immediate problem or topic. By stepping away from a scientific problem and exploring poetry, scientists can foster creativity through what is commonly referred to, in creativity research, as an *incubation period*. This is a process whereby initial conscious thought is followed by a period during which one refrains from task-related conscious thought (Gilhooly et al. 2013, Ritter and Dijksterhuis 2014). Incubation periods allow scientific ideas to percolate, and Aslan and colleagues (2014) discussed how incubation periods, or relaxed reflection, are an essential element of the creative process and highlight the importance of these periods for conservation scientists, who are likely to jettison periods of reflection in their working process. Importantly, incubation periods can illuminate hidden relationships, allowing for altered or changed views (Aslan et al. 2014), and drawing on activities dissimilar in nature to the target task, such as writing poetry, has been shown to have stronger effects on creative performance than an interpolated activity similar to the target task (Gilhooly et al. 2013). If creative performance and idea generation were the target of such incubation periods, conservation scientists could benefit from injecting an incubation period in their practice by writing on topics unrelated to the target task.

Integrating poetry as a component of daily writing could also assist conservation scientists with digesting and learning complex topics. Poetry, particularly shorter forms, such as haiku, can allow scientists to quickly capture and express new ideas. Pollack and Korol (2013) demonstrated the use of haiku as a means to convey neurobiological concepts succinctly by focusing on the most salient features of observed processes. Similarly, scientists can record creative impulses or intuitions related to a subject or problem without the constraints imposed by traditional scientific writing for journal publications. Breaking down thoughts on a topic in smaller, succinct thoughts or phrases could also help scientists to identify key themes or elements of a topic that need to be addressed or communicated clearly in their scientific writing or presentations.

The language and style of scientific publications are at times challenging to digest for subject experts, let alone other readers (Doubleday and Connell 2017). Poetry can offer a way for scientists to play with language, to reframe concepts, and to engage with aesthetics to capture readers in ways that are not possible with scientific articles (Silverman 2016). Zwart (2014) found that when asked to define nature and write a poem that captures nature, students’ poems proved more convincing than their definitions. In a related way, poems composed of words or phrases found in scientific articles could offer a way for scientists to engage with audiences who would otherwise not read such publications but who might read poetry or other short-form writing. For example, Madhur Anand writes poems composed of words and phrases found in her own scientific articles (Anand 2015). These *found* poems (poetry created from words or phrases taken from other sources) are a major component of Anand's debut book of poems, *A New Index for Predicting Catastrophes* (Anand 2015). Compilations of poetry such as those produced by Anand (2015) offer a unique form of expression and communication for scientists to engage and creatively communicate about a scientific topic. In an interview with Anand about the book, Follett (2016) said,

Here I’m thinking of your found poems which are created from phrases pulled from your published scientific articles. In order to better understand these poems, your readers may be pushed to develop their understanding of scientific concepts. Even more interesting is that by creating something new out of your previous publications, you are demonstrating that knowledge is always in process, even for experts. That knowledge is not fixed, or unattainable, is, I think, a reassuring realization for nonspecialist readers, and it may even invite them to create their own interpretations of your poems and put their gleaned scientific understanding to work against environmental injustices.

Through poetry, scientists can also potentially relate scientific topics to day-to-day activities or events and can transcend disciplinary boundaries to reach new audiences who might otherwise not be aware of or engaged with conservation issues. At the same time, as was highlighted by Follett (2016), many readers of Anand's poetry will have to embrace the discomfort associated with treading into new ideas or concepts, and the same could be true for scientists who read poems (not associated with science per se) and do not necessarily understand the concept or point being expressed. Embracing such discomfort can be beneficial in preparing the mind to take risks that lead to greater innovation, not only in poetry, but also in science.

**Figure 1.**
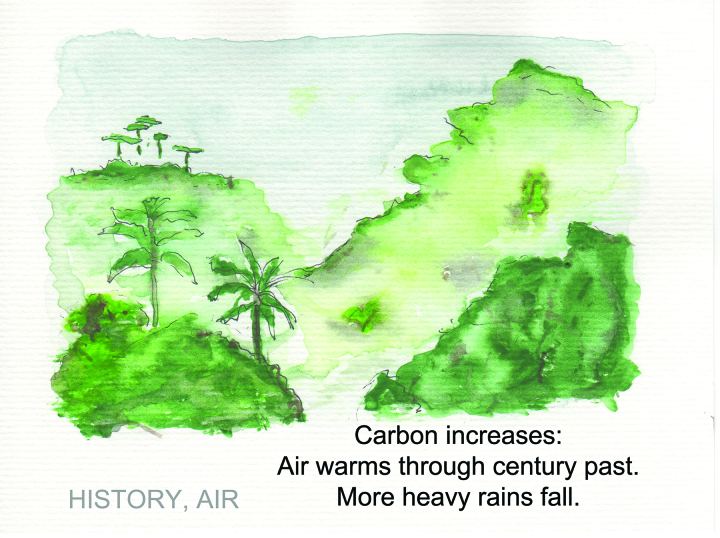
Haiku and image related to main topic in the IPCC State of the Climate in 2013 Executive Summary. Haiku and image by Gregory Johnson.

How, functionally, can poetry be used to distil peer-reviewed publications to create innovative approaches to communication and dialogue about complex topics? The process itself requires scientists to read peer-reviewed publications (e.g., journal articles), absorb the crucial information, and transform this knowledge into compact packages of information in poetic form. For example, Gregory Johnson, an oceanographer at the National Institute for Oceanic and Atmospheric Association and contributor to the Intergovernmental Panel on Climate Change (IPCC), used haiku to distil climate change science. Prior to a meeting to discuss the *IPCC State of the Climate in 2013* report—279 pages in length—Johnson was reviewing the *Summary for Policymakers*. In an attempt to organize his thoughts and pare down the summary, Johnson wrote haiku on the main topics covered within the summary (e.g., the atmosphere, sea levels, carbon cycles) that, when read together, told the narrative of the planet's changing climate. Johnson later painted watercolors to accompany the haiku (figure 1), which were covered by news outlets, such as *TheHuffington Post* (2013). Since then, Johnson has given lectures on his haiku and artistic process; incorporated haiku into the *IPCC State of the Climate* reports in 2014, 2015, and 2016; and published a paper (Johnson and Birnbaum 2017) with a haiku as the title:

As El Niño builds,

Pacific Warm Pool expands,

ocean gains more heat

Ultimately, as conservation scientists, we aim for our work to be understood and used by the individuals seeking or encountering it. Although scientists might have some reservations about poetry as a method of inquiry, when viewed more broadly, the affective nature of poetry can be a mechanism for users of scientific content to experience deeper learning and connection about otherwise unfamiliar topics. Through word play, sound, formal constraints, and aesthetics, poetry affectively engages the reader while effectively allowing the exploration of complex or unfamiliar topics.

## Building a science-poetry community

**Figure 2.**
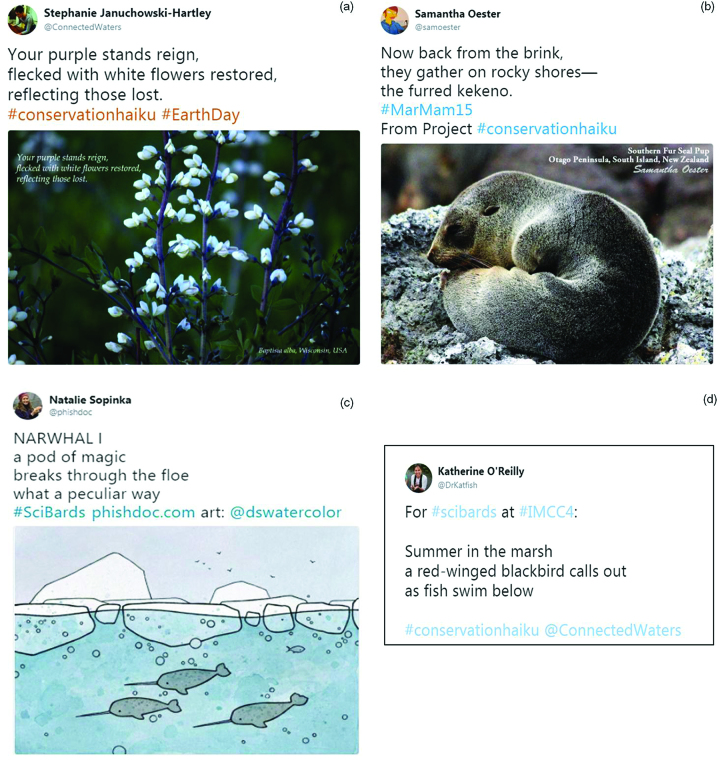
There is a need for a more inclusive definition of creativity in the science community. Scientists are (a) exploring the use of poetry to communicate about conservation and are using these efforts to (b) raise awareness about specific topics, programs, and initiatives. There is a growing (c) science-poetry community that is (d) encouraging conservation scientists to integrate poetry into their practice.

In 2015, Samantha Oester and Stephanie Januchowski-Hartley started a digital media project, Project Conservation Haiku, blending poetry, photography, and science together as a way to enhance people's engagement with conservation and the environment. A major component of Project Conservation Haiku was to connect science and poetry. The authors shared a unique haiku and photograph combination on Twitter (figure 2a and b), weekly, for a year. The project also developed into a blog in which the authors share more details about the inspiration and stories behind each poem that they wrote during the year of the project. The Project Conservation Haiku blog (*https://conservationhaiku.org*) offers a space in which readers can engage with the original haiku and photograph combinations and, if they are interested, can read a longer-form article that shares stories and knowledge about the topic covered in each haiku. Project Conservation Haiku not only brings environmental conservation issues to the fore through digital media, but it also brings poetry to others who, inspired by the project, spontaneously share their own haiku, such as this poem by Josh Silberg (shared using the project's hashtag, #ConservationHaiku, on Twitter):

Oh poor, poor ratfish

So chock full of parasites

Anus copepod.

The interest and uptake of conservation-inspired poetry because of Project Conservation Haiku further highlights the potential for poetry to enhance scientific engagement and learning.

Through Project Conservation Haiku, the authors also built collaborations with other scientists who write poetry related to conservation. These collaborations led to the formation of SciBards (#SciBards on Twitter), a small community of conservation scientists who write, read, speak, and share poetry (figure 2c and d). The SciBards community interacts primarily via social media, although individual and collaborative projects beyond social media have developed. For example, through the SciBards community, four of this article's coauthors, jointly facilitated a workshop at the 4th International Marine Conservation Congress to share their science-poetry experiences and bring poetry to conservation scientists. The “Using a pencilfish to write whales” workshop blended poetry, history, and science to address the relationship between science and poetry from several angles. At a conference in which science communication was a recurring theme, this workshop investigated a form not typically considered a science communication tool.

Through the “Using a pencilfish to write whales” workshop, several of this article's authors demonstrated how science and poetry can come back together and how engaging with poetry can improve conservation science. The workshop was opened with an introduction by Anna Zivian, performed in iambic quadrameter, presenting the inspiration for the poetry workshop. The use of poetry brought a sense of play to the workshop and demonstrated how poetry is well suited for reframing complex topics. The introduction was followed by a presentation from Natalie Sopinka on the history of science and poetry, which can be traced back to the earliest oral traditions. Her talk focused on the separation of science and poetry in the nineteenth century, when Victorian researchers exploring the natural world made an explicit shift from being *natural philosophers* to calling themselves *scientists*, a neologism by Whewell modelled after *artist* (Yeo 1993). This differentiation also led to the separation of poetry, which was previously joined seamlessly with natural philosophy, from “serious” science in the Anglophone world. To close, Sopinka highlighted the realignment of science and poetry—a recognition that the split has always been somewhat artificial. This was followed up by an applied example from Januchowski-Hartley about the reconnection of science and poetry through the digital media project that inspired the workshop. The workshop closed with several poetry exercises led by Oester. The exercises were focused on introducing the manifold purposes of poetry and poetry writing techniques to an audience with different levels of familiarity with the subject and connecting the practice of poetry with conservation science. The last presentation focused on form, feelings, function, and freedom, interspersing lessons about poetic technique with group exercises to highlight various aspects of poetry in a conservation context. In one exercise, participants wrote a found poem, reworked from an abstract in the conference program. Through this exercise, Josh Drew created the following found poem, drawn from an abstract written by Demian D. Chapman:

Cantonese delicacy

Shark

Fin

Soup

Significant global

Problem.

Reflecting on our experiences in the “Using a pencilfish to write whales” workshop, we determined that one way to grow a science-poetry community is to share examples and perspectives, such as those that we have set out in this article, from our own as well as others’ experiences with poetry and science–poetry integration. To further inspire conservation scientists to integrate poetry in their daily practice, we present two short poetry exercises. The first poetry exercise is adapted from Maxine Hong Kingston's *To Be the Poet* (2002), based on a method for writing poems that was shared with her by Ted Sexauer, a member of Kingston's Veterans’ Writing Workshop. The original exercise was adapted and expanded by Christina Lux (article coauthor) and is well suited for scientists with little to no background in creative writing.

**Step 1.** Close your eyes and become aware of your emotions or bodily sensations. Now, open your eyes and begin jotting down notes about what you observe in your immediate environment. Close your eyes again, sit with the emotion or feeling that emerges in your body; write it down and again observe your environment, jotting down your immediate impressions. Repeat this step until you believe you are done.

**Step 2.** Look back at your notes. What images or descriptions are most striking to you, which ones do you feel you might want to keep? Circle them. Begin a new draft, pulling from those circled ideas. Is a concept emerging? Consider this draft your *seed*. You can leave the seed and come back to it, or you can continue to develop it.

**Step 3.** Your seed contains patterns that you can now sharpen, uncover, and highlight, depending on the message you want to convey in the poem or the concept that you want to explore. Is there a pattern emerging from the words you’ve laid out on the page? Do you notice a cadence or rhythm in your draft? If you see a pattern emerging, think about the form that will best reflect or deepen the sense of the images in your poem, and further develop the writing.

**Step 4.** Choose what to do with the poem: share it on social media, publish it in a journal or magazine, read it at a poetry reading, pair it with the work of an artist, or keep it for yourself.

The second poetry exercise is drawn from Oester (2016) and from several exercises that we have led, or read about (e.g., Wolters and Wijnen-Meijer 2012). This second exercise offers scientists a framework to develop a short poem about their research, a question, or broader ideas about science practice.

First, identify your muse: an objective, a topic, or an experience that you want to write about. Now think about and even write down what you are trying to accomplish. Write down everything you can think of about your muse, inspiration, experience, or story. Don’t edit, just write. Set down the writing and thoughts for at least an hour. Return to the writing, read it over, and begin to clarify the idea that you are trying to accomplish or convey. Develop line breaks in your writing to create a juxtaposition, to evoke sensation or drive a narrative forward. Edit as needed, potentially ruthlessly, until you have a poem you are satisfied with, one that aligns with whatever type of poem you want to create (e.g., haiku, sonnet, or limerick). Consider sharing your work with others for reactions and feedback. Potentially revisit your poem again, and revise as you see best. Finally, reflect on your poem, and share with others if you wish.

A more detailed overview of poetry and the basics of writing it are set out in a short course by Oester (2016). Such exercises can be used by scientists to further develop or refresh their poetry writing. We encourage scientists to draw on these resources and to share their poetry and creative processes with others—scientists and nonscientists alike.

## Conclusions

We support recent calls from Aslan and colleagues (2014) and Zavaleta and colleagues (2017) for a broader and more inclusive definition of creativity to be promoted in the conservation science community and in other scientific communities more broadly. There is considerable evidence that exercising creativity through poetry writing, reading, or speaking can develop, maintain, and enhance empathic and innovation skills. Integrating creative practices, such as poetry writing, and developing these skills should be essential components of professional development and practice of conservation scientists. Accordingly, whether in the office, lab, or field, writing and sharing poetry can foster creativity and enhance conservation scientists’ engagement and learning of unfamiliar topics. We base this assertion on the benefits and opportunities detailed in the literature and on those we have observed in our own interdisciplinary practices and projects that integrate poetry with conservation science. Additional benefits to conservation science and practice derived from poetry integration could likely be elucidated through additional work on this topic. Indeed, the potential benefits of science–poetry integrations remain poorly explored (although the effectiveness of poetry as a science communication tool is being investigated; Illingworth 2016). Opportunity exists to grow projects and further our understanding of what such initiatives can offer. Identifying approaches that effectively bring together diverse perspectives and tools to inject creativity into complex problems will strengthen our ability to overcome some of society's toughest challenges (NASEM 2018).

## Author Biographical

Stephanie R. Januchowski-Hartley (s.r.januchowski@swansea.ac.uk) is affiliated with the Department of Biosciences at Swansea University, in Swansea, United Kingdom. Natalie Sopinka is affiliated with Canadian Science Publishing, in Ottawa, Ontario. Bethann G. Merkle is affiliated with the Wyoming Migration Initiative, in the Department of Zoology and Physiology at the University of Wyoming, in Laramie. Christina Lux is affiliated with the Center for the Humanities at the University of California, Merced. Anna Zivian is affiliated with the Ocean Conservancy, in Santa Cruz, California. Patrick Goff is affiliated with Beaumont Middle School, in Beaumont, Kentucky. Samantha Oester is affiliated with the Environmental Science and Policy Department at George Mason University, in Fairfax, Virginia.
